# Pulmonary colloid adenocarcinoma mimicking lung abscess with concurrent KRAS and TP53 mutations: a case report

**DOI:** 10.3389/fmed.2025.1749324

**Published:** 2026-01-20

**Authors:** Green Hong, Yooyoung Chong, Joo-Eun Lee, Mi Jung Lim, Chaeuk Chung, Da Hyun Kang

**Affiliations:** 1 Department of Internal Medicine, College of Medicine, Chungnam National University, Daejeon, Republic of Korea; 2 Department of Thoracic & Cardiovascular Surgery, College of Medicine, Chungnam National University, Daejeon, Republic of Korea; 3 Department of Biomedical Research Institute, Chungnam National University Hospital, Daejeon, Republic of Korea; 4 Genomics Department, Keyomics Co. Ltd., Daejeon, Republic of Korea

**Keywords:** case report, differential diagnosis, KRAS andTP53 mutations, lung abscess, pulmonary colloid adenocarcinoma

## Abstract

**Background:**

Pulmonary colloid adenocarcinoma is a rare subtype of lung adenocarcinoma, representing fewer than 1% of all cases. Characterized by abundant extracellular mucin and sparse tumor cells, it frequently mimics infectious lung diseases, making preoperative diagnosis particularly challenging.

**Case presentation:**

We report the case of a 44-year-old woman who presented with fever, pleuritic chest pain, and dyspnea. Laboratory evaluation revealed leukocytosis, elevated C-reactive protein, and increased procalcitonin. Radiologic findings showed a large, septated cystic lesion measuring 11 cm in the right lower lobe, accompanied by lobar consolidation and a small pleural effusion, initially suspected to be a lung abscess. Despite the administration of antibiotics and attempted percutaneous drainage, symptoms persisted. Consequently, surgical resection was performed, and histopathology confirmed colloid adenocarcinoma with visceral pleural invasion but no nodal metastasis. The patient subsequently received adjuvant chemotherapy and has remained recurrence-free for over 3 years. Targeted next-generation sequencing identified co-occurring KRAS and TP53 mutations along with a truncating AXIN2 variant, suggesting concurrent activation of the RAS/MAPK and Wnt/β-catenin pathways.

**Conclusion:**

Pulmonary colloid adenocarcinoma can clinically and radiologically mimic a lung abscess. Failure to respond to standard antibiotic therapy and drainage should prompt consideration of an underlying malignancy. Surgical resection is essential for both definitive diagnosis and treatment.

## 1 Introduction

Pulmonary colloid adenocarcinoma is an extremely rare histologic subtype of lung adenocarcinoma, accounting for <1% of all cases. Histologically, it is characterized by abundant extracellular mucin pools containing scattered clusters of malignant epithelial cells (1, 2). Diagnosis is often delayed or missed due to its rarity and atypical features. Radiologically, colloid adenocarcinoma typically presents as a uni- or multilocular, cystic, and lobulated, low-attenuation lesion with septal or mural enhancement and stippled calcifications on contrast-enhanced computed tomography (CT). These features can closely resemble those of various infectious or inflammatory conditions (3).

In contrast, a lung abscess is a relatively common condition resulting from necrosis and cavitation of lung parenchyma secondary to infection. Patients typically present with fever, productive cough, pleuritic chest pain, and systemic inflammatory response. Imaging often reveals cavitary or septated cystic lesions with fluid levels and surrounding consolidation. Common predisposing factors include underlying structural diseases, poor dental hygiene, and chronic alcohol use. In most cases, appropriate intravenous antibiotics with or without percutaneous drainage achieve satisfactory resolution. Surgical resection is generally reserved for patients with persistent infection despite several weeks of medical therapy, severe complications such as uncontrolled sepsis, or suspected underlying structural abnormalities (4–6).

Because of these diagnostic challenges, malignant tumors that mimic lung abscesses are rare but clinically significant. Failure to respond to standard therapy should prompt consideration of alternative diagnoses, including uncommon neoplasms.

Here, we report a rare case of pulmonary colloid adenocarcinoma that closely mimicked a lung abscess on both clinical and radiologic evaluations. This case emphasizes the need to consider uncommon malignancies in the differential diagnosis of lung abscess–like lesions unresponsive to conventional treatment.

## 2 Case presentation

A 44-year-old woman presented with a 1-week history of chest discomfort and dyspnea. Three days before admission, her symptoms worsened, and she developed right-sided pleuritic chest pain, cough, and sputum production. She had no significant medical history but reported binge drinking (two bottles of soju once weekly).

**Figure 1:**
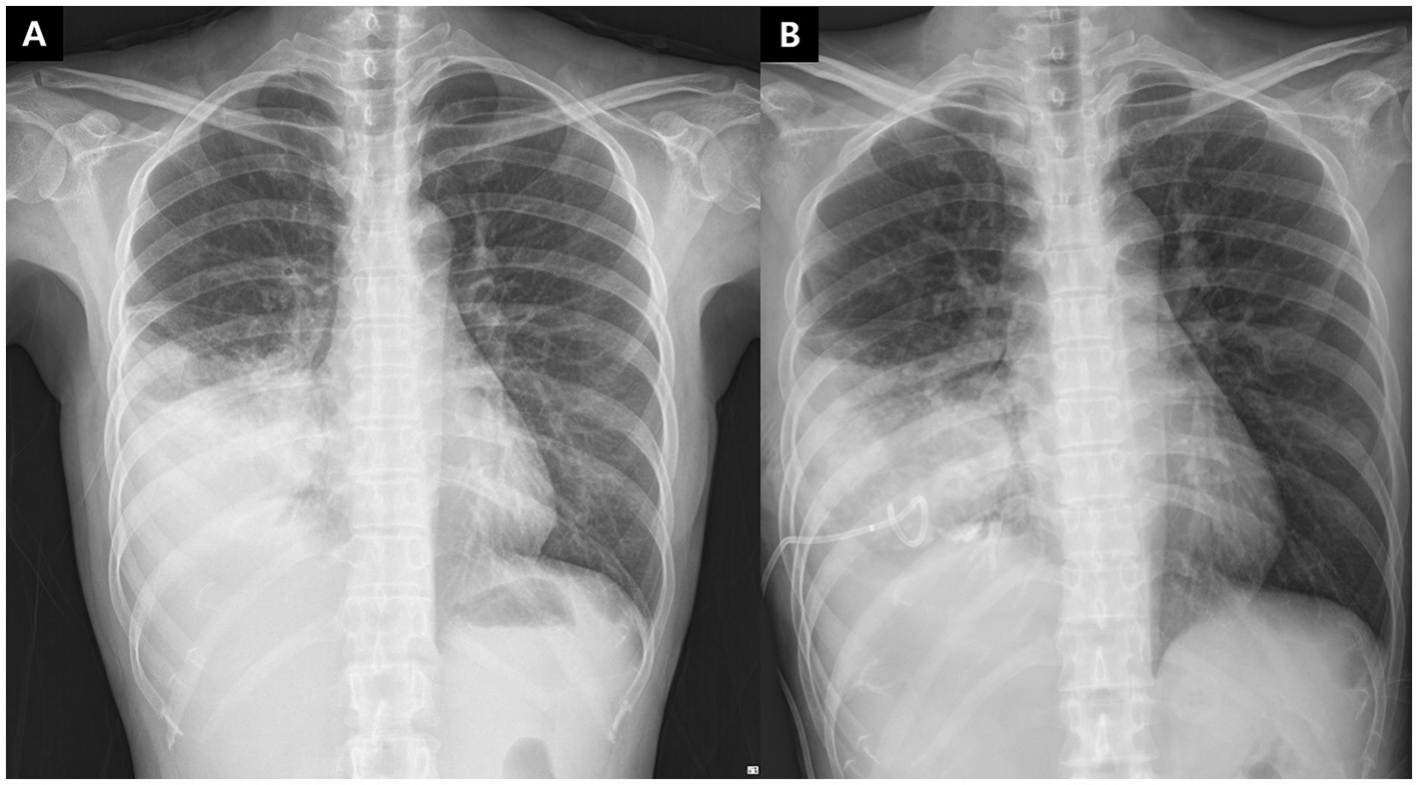
Chest radiographs of the patient. **(A)** Initial chest X-ray at admission. **(B)** Chest x-ray 3 days after the initial visit, following percutaneous catheter drainage insertion.

**Figure 2:**
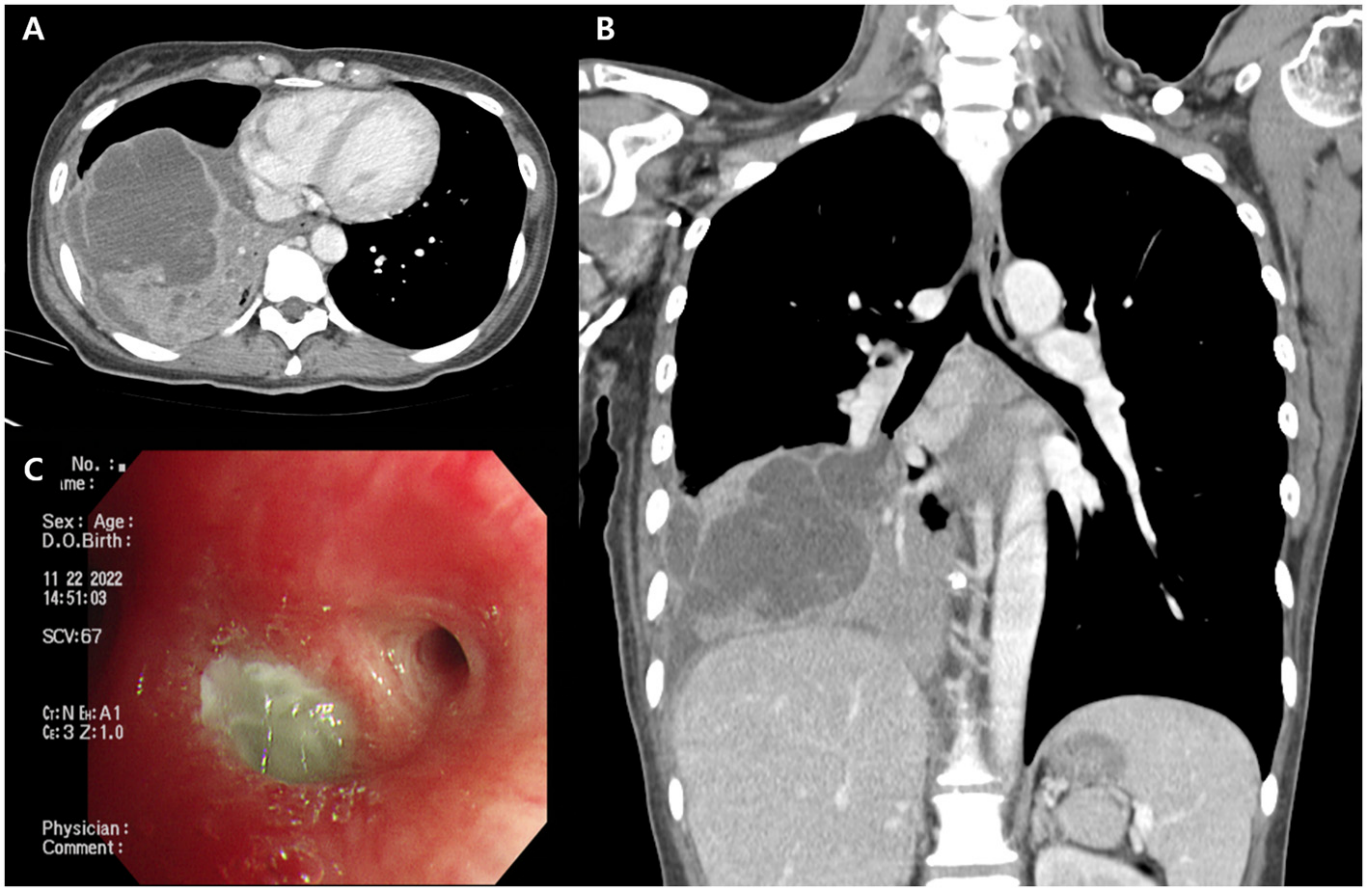
Radiologic and bronchoscopic findings of pulmonary colloid adenocarcinoma. **(A)** Axial contrast-enhanced CT image showing a well-defined, multilocular cystic lesion with thin septal and mural enhancement in the right lower lobe. **(B)** Coronal view demonstrating the multilocular cystic architecture and enhancing septa. **(C)** Bronchoscopy imaging showing the right lower lobe basal segmental orifice covered with abundant mucin.

On admission, she was febrile (temperature: 38.7 °C) and tachycardic (heart rate: 127 beats/min). Physical examination revealed a productive cough and right-sided pleuritic chest pain. Laboratory investigations showed leukocytosis (white blood cell count: 16,900/μl), elevated C-reactive protein (> 8.0 mg/dl), and increased procalcitonin (1.01 ng/ml). Chest radiography revealed right lower-lobe consolidation (Figure 1A). Chest radiography and CT revealed lobar consolidation with airspace opacities and endobronchial secretions, as well as a 9-cm lobulated, septated cystic lesion in the right lower lobe, initially interpreted as a lung abscess (Figures 2A, B). The lesion exhibited heterogeneous low attenuation with internal septations and peripheral enhancement, findings frequently observed in necrotizing infection or abscess formation. It showed thin but irregular wall enhancement and subtle internal septations, without the thick rim enhancement or peripheral edema typically seen in acute abscess formation, making radiologic differentiation from a mucin-producing neoplasm challenging. A small volume of pleural fluid with smooth, enhancing pleural walls was also observed, raising suspicion for associated empyema.

The patient was administered intravenous antibiotics for presumed lung abscess and possible empyema. Bronchoscopy revealed thick mucopurulent secretions obstructing the right lower lobe bronchial orifice, which were subsequently removed (Figure 2C). After clearance of the secretions, only mucosal edema was observed, without evidence of an endobronchial lesion. However, after 3 days of antibiotic therapy, the fever persisted above 38 °C. Percutaneous catheter drainage was attempted, but no fluid was aspirated (Figure 1B). Given the absence of clinical improvement, surgical intervention was planned for a presumed lung abscess.

**Figure 3:**
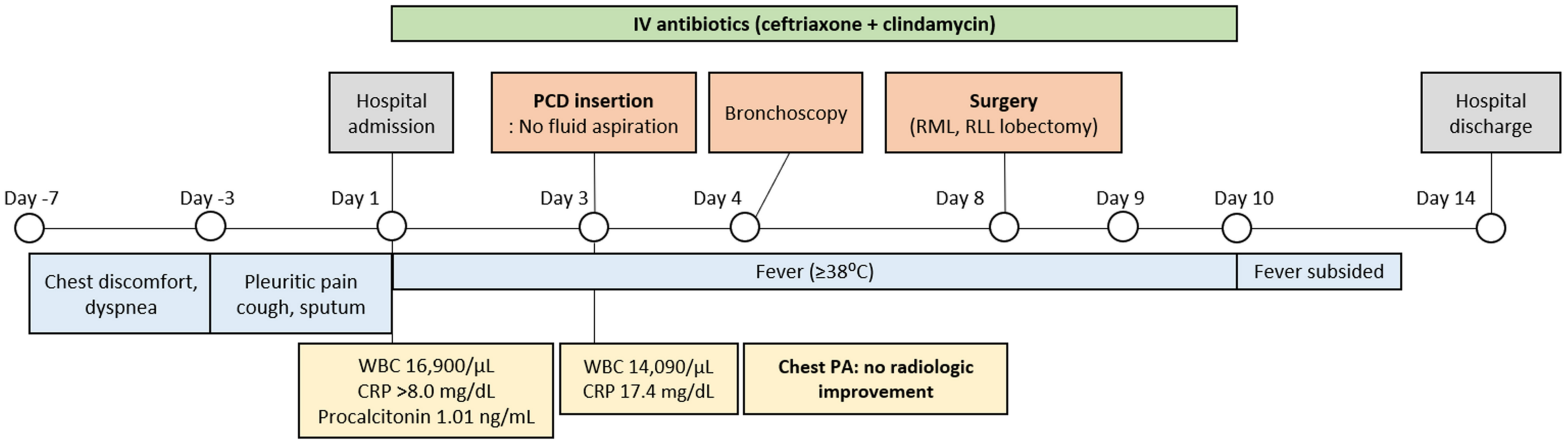
Clinical timeline of the patient's diagnostic and therapeutic course. The timeline summarizes symptom onset, laboratory findings, radiologic evaluation, intravenous antibiotic treatment, percutaneous catheter drainage attempt, surgical intervention, and postoperative outcome. Serial chest radiographs demonstrated no radiologic improvement despite appropriate antibiotic therapy, leading to definitive surgical management.

Video-assisted thoracoscopic surgery was initially performed for right lower lobectomy, but was converted to thoracotomy due to a huge consolidative mass involving the right lower lobe. Intraoperatively, a 2 cm-sized defect was identified in the lateral basal segmental bronchus, with discharge suggestive of mucus impaction. The right middle lobe also showed severe consolidative changes; therefore, to minimize the risk of additional postoperative bronchopleural fistula, a right middle and lower bilobectomy was performed. Intraoperative frozen section analysis was not requested because the lesion was strongly suspected to be infectious. The postoperative course was uneventful, and the patient was discharged on postoperative day 6 without complications. The overall clinical course and key diagnostic and therapeutic milestones are summarized in a clinical timeline (Figure 3).

**Figure 4:**
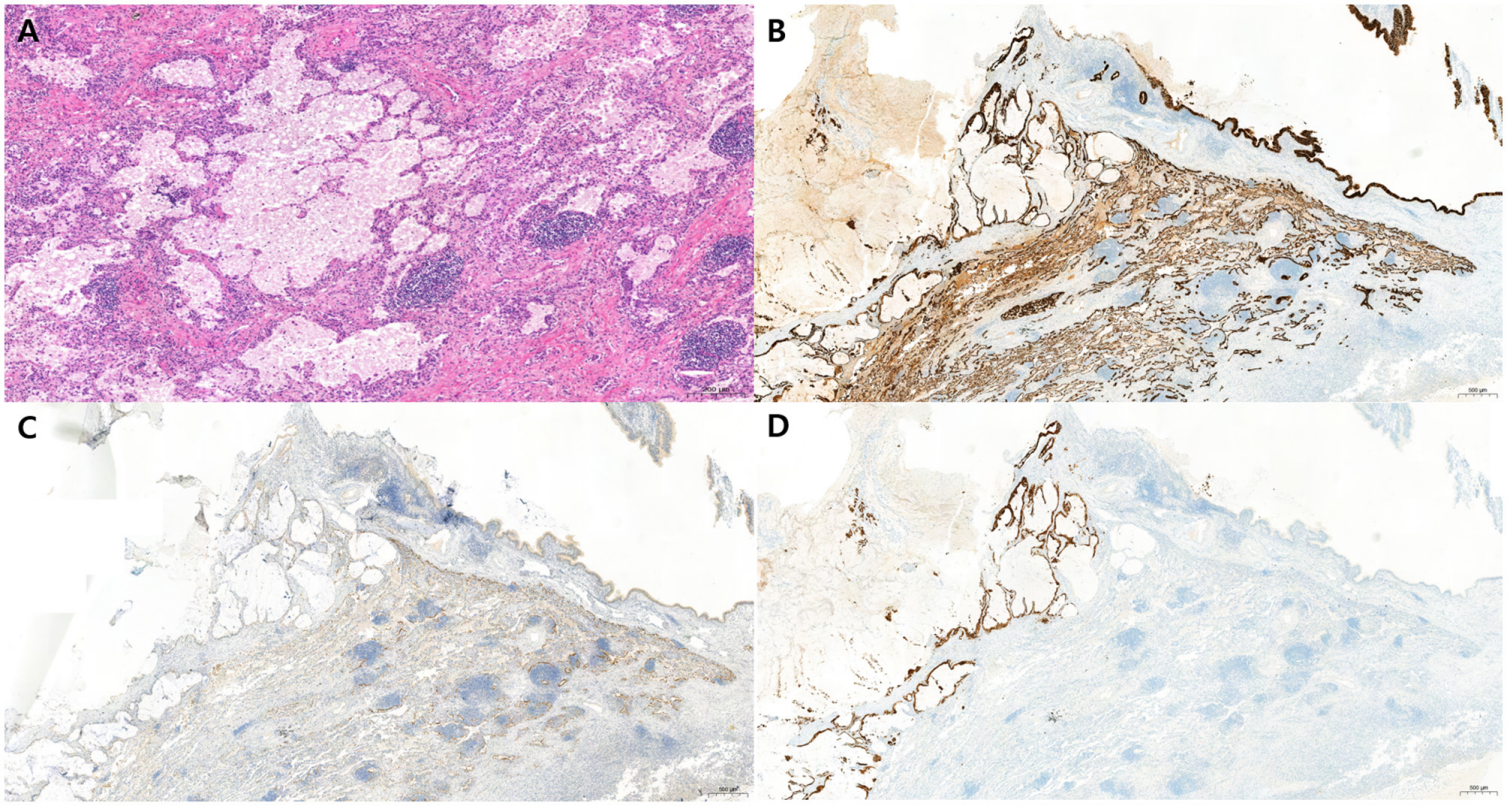
Histopathologic features of pulmonary colloid adenocarcinoma. **(A)** Hematoxylin and eosin (H&E) staining showing large extracellular mucin pools containing floating clusters of neoplastic epithelial cells exhibiting mild to moderate nuclear atypia. The mucin pools are separated by fibrous septa and accompanied by inflammatory cell infiltration. (Original magnification, ×100; scale bar = 200 μm). **(B)** CK7 staining showing positivity. **(C)** CK20 staining also shows positivity. **(D)** TTF-1 staining showing negativity. (Original magnification, ×40; scale bar = 500 μm).

Histopathological examination unexpectedly revealed colloid adenocarcinoma measuring 11.0 × 9.3 × 7.3 cm, with visceral pleural invasion classified as PL2 (Figure 4A). The surrounding lung parenchyma of the right middle and lower lobes showed acute and chronic inflammation with abscess formation. Examination of the surgical specimens and postoperative imaging studies showed no evidence of mediastinal lymph node or distant organ metastases.

Immunohistochemical staining demonstrated positivity for CK7 (Figure 4B) and CK20 (Figure 4C), focal positivity for CDX2, and negativity for TTF-1 (Figure 4D). EGFR mutation testing and ALK rearrangement were both negative, and PD-L1 expression (sp263) had a tumor proportion score of 0%. Targeted DNA/RNA next-generation sequencing (solid tumor panel including TMB/MSI) identified no Tier 1 (strong clinical significance) variants. Tier 2 variants included KRAS p.G12V (c.35G>T), TP53 p.R273H (c.818G>A), and CTNNB1 p.S37F (c.110C>T). A Tier 3 variant of uncertain significance was detected in SMAD4 p.D537G (c.1610A>G). The tumor mutational burden (TMB) was 4.74 mutations/Mb, and the microsatellite status was stable (MSS).

**Figure 5:**
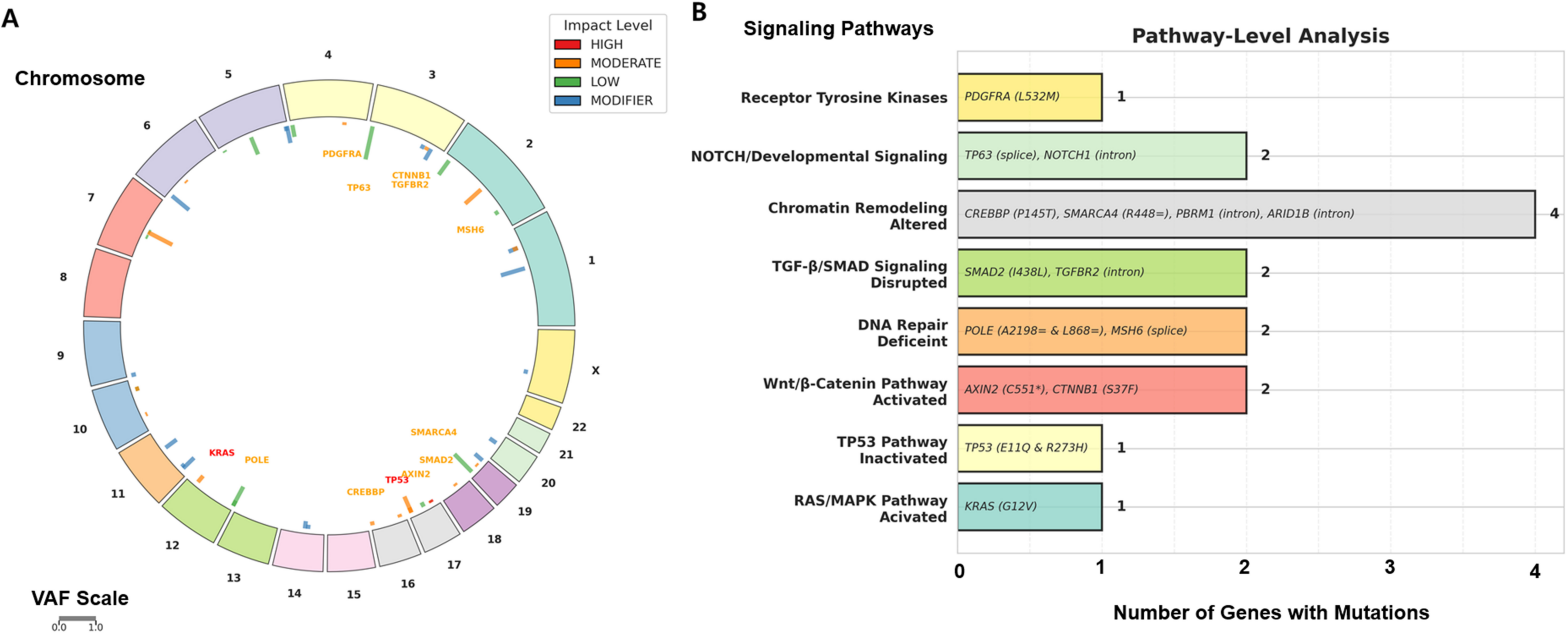
Somatic mutation landscape and pathway-level analysis of colloid adenocarcinoma. **(A)** Circular plot illustrating the chromosomal distribution and predicted impact of somatic mutations identified by re-analysis of Ion Torrent targeted next-generation sequencing data. Variants are color-coded by predicted impact (red, high; orange, moderate; green, low; blue, modifier). Key driver mutations include KRAS p.G12V, TP53 p.R273H and p.E11Q, CTNNB1 p.S37F, and AXIN2 truncating mutation. **(B)** Pathway-level summary of affected signaling networks, highlighting concurrent activation of the RAS/MAPK and Wnt/β-catenin pathways and inactivation of the TP53 pathway. Collectively, these alterations suggest a cooperative genomic background contributing to the mucin-rich colloid phenotype of the tumor.

Subsequent bioinformatics re-analysis of the targeted next-generation sequencing raw data was performed using the original Ion Torrent reads to explore potential additional variants beyond those initially reported. The raw data, initially aligned to hg19, were re-mapped to the hg38 reference genome, and somatic single-nucleotide variants were identified using Mutect2 with stringent filtering. This re-analysis revealed additional mutations, including TP53 p.E11Q and a truncating AXIN2 variant, indicating activation of the RAS/MAPK and Wnt/β-catenin pathways and inactivation of the TP53 pathway (Figure 5).

Given the pathologic stage (T4N0) with visceral pleural invasion (PL2), adjuvant chemotherapy with cisplatin and vinorelbine for four cycles was administered. The patient has remained disease-free for over 3 years of follow-up.

## 3 Discussion

Pulmonary colloid adenocarcinoma is an exceptionally rare subtype of invasive adenocarcinoma, representing <1% of primary lung carcinomas (7–10). Histologically, it is defined by abundant extracellular mucin pools containing sparse neoplastic cells floating within, often resulting in alveolar wall destruction (7, 9). Immunohistochemically, tumor cells typically express CK7, with variable CK20 and CDX2 positivity, which can mimic gastrointestinal mucinous adenocarcinomas, making the exclusion of metastatic disease essential (7, 9, 10).

Preoperative diagnosis of pulmonary colloid adenocarcinoma is often challenging because its radiologic features are nonspecific. Previously reported imaging findings include lobulated cystic or cavitary masses with low attenuation and minimal enhancement on CT, high signal intensity on T2-weighted Magnetic resonance imaging (MRI), and low or heterogeneous uptake on Fluorodeoxyglucose Positron Emission Tomography (FDG-PET) (3, 7, 8). The predominance of mucin and paucity of malignant cells frequently render transbronchial or needle biopsy specimens non-diagnostic, necessitating surgical resection for definitive diagnosis (3, 9).

Several studies have emphasized that non-diagnostic bronchoscopy or repeated biopsies yielding only mucin should raise suspicion for mucinous neoplasms such as colloid adenocarcinoma, particularly when clinical or radiologic findings are atypical for simple infection. Most published cases have described incidental or indolent tumors, even when large (Table 1). For instance, Ogusu et al. (7) reported an asymptomatic 76-year-old man with a cystic mass in the right lower lobe, in whom both CT and MRI suggested a mucin-rich lesion; however, the preoperative biopsy was inconclusive, and the diagnosis of colloid adenocarcinoma was established only after resection. Similarly, Nakamura et al. (8) described a giant 11-cm tumor resected via median sternotomy in a 69-year-old woman who remained asymptomatic until diagnosis, highlighting that even large colloid adenocarcinomas may remain clinically silent. Other reported cases have described lesions presenting either as irregularly marginated masses or well-defined nodules; however, these tumors were likewise asymptomatic, and transbronchial lung biopsy or CT-guided biopsy failed to demonstrate definitive malignant cells, with surgical resection ultimately required for both diagnosis and treatment (11–13). Only rare reports have described colloid adenocarcinoma in association with overt infection, such as concomitant pulmonary actinomycosis, rather than presenting as an isolated neoplastic process (14).

**Table 1:** Reported cases of pulmonary colloid adenocarcinoma and malignant lung tumors mimicking lung abscess.

**Year**	**Age/sex**	**Size (mm)**	**Location**	**Clinical symptom**	**Radiologic finding**	**Histology**	**Stage**	**Diagnosis, treatment, and outcome**	**Reference**
2018	76/M	55	RLL	None	Low attenuate cystic mass lesion with delayed slight septal enhancement	Colloid adenocarcinoma	pT3N0M0	TBLB: No malignant cell Surgery	(7)
2020	69/F	115 × 90	RUL	None	A giant cystic mass with calcification and contrast effects in the cystic wall	Colloid adenocarcinoma	pT4N0M0	Surgery Postoperative adjuvant chemotherapy For 1.5 years postoperatively without recurrence	(8)
2022	63/M	23	LUL	Dyspnea	Irregular perifissural nodule with spiculated contours and a central necrosis	Colloid adenocarcinoma	pT2N0M0	Surgery For 2 year without recurrence	(9)
2023	66/F	55 × 40	LLL	None	Large mass with slightly irregular margins The interior of the mass was a mixture of cyst-like hypodense areas and hyperdense areas	Colloid adenocarcinoma	pT3N0M0	Surgery For 4 years postoperatively without recurrence.	(11)
2024	68/M	23 × 21	RUL	None	Well-defined, low attenuation nodule	Colloid adenocarcinoma	pT1bN0M0	TBLB: No malignant cell Surgery Recur after 1 year	(12)
2025	77/M	35 × 30	RLL	None	Irregular subpleural mass surrounded by a halo of consolidation	Colloid adenocarcinoma	pT2N0M0	CT-guided biopsy revealed abundant mucin pools with few atypical cells. Surgery	(13)
2025	66/M	41	RUL	Cough, bloody sputum	Lobulated mass filled with soft tissue density area, accompanied by peripheral consolidation	Colloid adenocarcinoma And pulmonary actinomycosis	pT3N0M0	TBLB: adenocarcinoma Surgery, adjuvant chemotherapy	(14)
2014	64/M	59 × 49	RUL	Cough, fever	Low-density lesion with thick ring-enhanced irregular walls, poorly defined margins	Pleomorphic carcinoma	pT2N0M0	TBLB and CT-guided fine-needle biopsy: inflammatory cells compatible with active inflammation, No malignant cell Surgery	(15)
2021	36/F	91 × 67	LUL	Cough, bloody sputum, fever	Circular enhancement mass like lesion with bubbles and liquid dark areas	Choriocarcinoma	IV	CT guided lung biopsy: no malignant cell Surgery	(16)
2023	35/F	95 × 74	RLL	Chest pain, fever, cough, hemoptysis	Multilobulated mass with central necrosis and air-filled spaces with multiple internal septa	Squamous cell carcinoma	pT4N0M0	CT-guided biopsy: extensively necrotic non-small cell carcinoma that showed keratinizing squamous differentiation Surgery and adjuvant chemotherapy Follow-up after 6 months showed no recur	(17)
2024	78/M	NA	LLL	Cough, sputum	Infiltration, ground glass opacities and bronchiectasis in the right upper and left lower lobes, multiple cavitary lesions scattered throughout both lungs, and an abscess cavity in the left lower lobe	Ciliated adenocarcinoma	cT4N1M1a	Bronchoscopic biopsy: ciliated adenocarcinoma	(18)

M, male; F, female; RUL, right upper lobe; RLL, right lower lobe; LUL, left upper lobe; LLL, left lower lobe; TBLB, transbronchial lung biopsy; CT, computed tomography; NA, not available.

Tumor stage and size were recorded as reported in the original case reports. Tumor size is presented as the maximum diameter when a single value is provided, and as the longest × shortest diameter when two values are available.

In contrast, our patient presented acutely with fever, dyspnea, pleuritic chest pain, leukocytosis, and elevated levels of inflammatory markers, strongly suggesting an infectious etiology. Her history of habitual alcohol consumption (approximately two bottles of soju per week) further supported the suspicion of a lung abscess. Radiologic findings showed a septated cystic lesion similar to a lung abscess with a small pleural effusion; however, percutaneous drainage yielded no fluid. To our knowledge, cases of pulmonary colloid adenocarcinoma presenting as a clinically refractory lung abscess are exceedingly rare, underscoring the potential for misdiagnosis. Nevertheless, several malignant lung tumors have been reported to mimic lung abscess or necrotizing pneumonia, leading to delayed diagnosis (Table 1). These include cases of pleomorphic carcinoma, choriocarcinoma, squamous cell carcinoma, and ciliated adenocarcinoma presenting with cavitary or necrotic lesions initially managed as infection (15–18). Notably, in some of these reports, CT-guided or transbronchial biopsies yielded negative or non-diagnostic results, and definitive diagnoses were established only after surgical resection (15, 16). In our patient, percutaneous biopsy was not initially considered because the lesion was presumed infectious, and sampling a large necrotic cavity carries procedural risks—such as infection spread or pneumothorax—and a low diagnostic yield. Surgical resection was ultimately performed after antibiotic therapy and percutaneous drainage failed, providing both therapeutic benefit and a definitive diagnosis. In retrospect, although earlier tissue confirmation might have accelerated management, percutaneous sampling would likely have remained non-diagnostic given the extensive mucinous and necrotic components. This case underscores the importance of timely surgical evaluation and consideration of malignant etiologies in non-resolving pulmonary lesions, particularly when patients fail to respond to appropriate antibiotic therapy despite clinical and laboratory findings strongly suggesting infection.

Because of its rarity, large-scale clinical data on pulmonary colloid adenocarcinoma are limited, and most available reports are individual case studies or small case series (9, 11–13). Surgical resection remains the mainstay of treatment, following the principles applied to conventional adenocarcinoma, and serving both diagnostic and therapeutic purposes (8, 9). Tumor size varies widely, from small peripheral nodules to giant tumors exceeding 10 cm (8, 10). Our patient's tumor measured 11 cm, similar to previously reported giant tumors resected successfully (8).

Notably, in colloid adenocarcinoma, it is often difficult to precisely determine the extent of invasion because the tumor frequently contains extensive mucinous areas with only focal epithelial proliferation. Consequently, the gross tumor size may overestimate the true invasive tumor burden. Despite the large tumor size and visceral pleural invasion (PL2), our patient has remained recurrence-free for over 3 years following surgery and adjuvant chemotherapy. This outcome is consistent with previous reports indicating that complete resection often yields favorable long-term survival (7, 10, 11), although local recurrence and distant metastases have also been reported (9, 10).

From a molecular standpoint, our case demonstrated co-occurring KRAS (p.G12V) and TP53 (p.R273H/E11Q) mutations, along with a truncating AXIN2 alteration identified by WES re-analysis. These findings imply concurrent activation of the RAS/MAPK and Wnt/β-catenin pathways and inactivation of the TP53 pathway, offering potential mechanistic insight into the mucin-rich phenotype characteristic of colloid adenocarcinoma. Although KRAS–TP53 co-mutations are relatively common in conventional lung adenocarcinomas, they have rarely been reported in the colloid subtype, making this case molecularly distinctive. Clinically, KRAS–TP53 co-mutation has been associated with unique biological and therapeutic implications. Several large cohorts have shown that this genotype may confer poorer prognosis than KRAS mutation alone, particularly in resected or mucinous adenocarcinoma subsets, suggesting a need for closer postoperative surveillance (19). However, in the context of immunotherapy, KRAS–TP53 tumors often exhibit an inflamed immune microenvironment characterized by higher tumor mutational burden and PD-L1 expression, and thus tend to show better responsiveness to immune checkpoint inhibitors compared with KRAS–STK11 or KRAS-only tumors (20). For KRAS-targeted therapies such as sotorasib or adagrasib, TP53 co-mutation alone has not been linked to primary resistance, unlike STK11 or KEAP1 alterations (21). Overall, these observations indicate that KRAS–TP53 co-mutant lung cancers represent a biologically active subtype with potentially aggressive behavior but preserved immunogenicity, suggesting that patients with this genotype might benefit from multimodal approaches combining targeted therapy and immunotherapy, along with closer surveillance for early recurrence.

This case has several strengths, including comprehensive correlation of radiologic findings with pathologic and genomic analyses, as well as long-term follow-up demonstrating durable disease control. However, as a single case report, the findings are inherently limited in generalizability, and radiologic features of pulmonary colloid adenocarcinoma may overlap with those of infectious or inflammatory lung diseases, potentially limiting preoperative diagnostic certainty.

In summary, pulmonary colloid adenocarcinoma exhibits a broad clinical spectrum, from incidental asymptomatic nodules to giant tumors or even infection-mimicking lesions, as in our case. Awareness of this variability is crucial, as failure to respond to standard antibiotic therapy or drainage in presumed lung abscesses should prompt consideration of rare malignant etiologies. Early surgical intervention not only provides a definitive diagnosis but also offers the best chance for long-term disease control.

## 4 Patient perspective

The patient initially believed the symptoms were due to an infection such as pneumonia and was shocked when informed that the lesion was cancer. Nevertheless, the patient expressed great relief that the tumor could be completely removed surgically at that stage, especially given her young age and the relatively large size of the mass.

## Data Availability

The datasets presented in this article are not readily available because they contain sensitive patient information. Requests to access the datasets should be directed to the corresponding author.
